# Supplementary material for: Fed-Batch Fermentation of Saccharomyces pastorianus with High Ribonucleic Acid Yield
Source: Foods. 2022 Sep 7;11(18):2742. doi: 10.3390/foods11182742 (PMC9497889; doi:10.3390/foods11182742)
Supplement: Supplementary file 1 [file foods-11-02742-s001.zip › foods-1845936-supplementary.pdf]

foods

Supplementary Materials

Table S1. Gas production ability of G03 and G03H8.

TIME/h	Gas production	
	G03	G03H8
4	-	-
6	-	+
8	+	++
10	+++	+++
11	++++	++++

No gas production: -; 0-1/3 tube volume: +; 1/3-2/3 tube volume: ++; 2/3-100% tube volume: +++; 100% tube volume: ++++.

Table S2. Statistics of SNP annotation results using *S.pastorianus*.CBS.1483 as the reference sequence.

Sample	Type	Start syn	Stop syn	Start non-syn	Stop non-syn	Premature stop	Synonymous	Nonsynonymous	Total CDS
G03	Number	0	0 5	5	16	45	3，616	3，007	6，694
	Rate/%	0.0000	0.0443	0.0443	0.1417	0.3987	32.0340	26.6389	59.3019
G03H8	Number	0	4	6	16	42	3，558	2，867	6，493
	Rate/%	0.0000	0.0367	0.0550	0.1467	0.3851	32.6213	26.2859	59.5306

Table S3. Results of the PB design.

Run	Molasses/%	$(NH_4)_2SO_4$/%	$MgSO_4 \cdot 7H_2O$/%	KH_2PO_4/%	$FeSO_4 \cdot 7H_2O$/(g/L)	$ZnSO_4 \cdot 7H_2O$/(g/L)	Yeast extract/%	RNA/ (mg/g DCW)
1	2	1	0.3	0.3	0.10	0.02	1.5	146.3
2	4	3	0.3	0.1	0.10	0.10	0.5	143.6
3	2	3	0.1	0.1	0.02	0.10	1.5	143.1
4	4	1	0.3	0.3	0.02	0.10	0.5	154.5
5	4	1	0.1	0.1	0.10	0.10	1.5	148.9
6	4	3	0.1	0.3	0.02	0.02	0.5	153.0
7	2	3	0.3	0.3	0.02	0.10	1.5	144.0
8	2	1	0.1	0.1	0.02	0.02	0.5	147.9
9	4	3	0.1	0.3	0.10	0.02	1.5	150.9
10	2	3	0.3	0.1	0.10	0.02	0.5	143.3
11	2	1	0.1	0.3	0.10	0.10	0.5	148.7
12	4	1	0.3	0.1	0.02	0.02	1.5	148.6

Table S4. Experimental design and results of the steepest ascent path.

	Molasses /%	$(NH_4)_2SO_4$/%	KH_2PO_3/%	$MgSO_4 \cdot 7H_2O$/%	$FeSO_4 \cdot 7H_2O$/(g/L)	$ZnSO_4 \cdot 7H_2O$ /(g/L)	Yeast extract /%	RNA/ (mg/g DCW)
1	1	4	0.10	0.2	0.06	0.06	1	119.0
2	1.5	3.5	0.15	0.2	0.06	0.06	1	108.6
3	2	3	0.20	0.2	0.06	0.06	1	121.3
4	2.5	2.5	0.25	0.2	0.06	0.06	1	143.3
5	3	2	0.30	0.2	0.06	0.06	1	116.8
6	3.5	1.5	0.35	0.2	0.06	0.06	1	137.2
7	4	1	0.40	0.2	0.06	0.06	1	155.7
8	4.5	0.5	0..45	0.2	0.06	0.06	1	148.1

Table S5. Experimental design and results of the central composite design.

	Molasses %	$(NH_4)_2SO_4$/%	KH_2PO_3/%	$MgSO_4 \cdot 7H_2O$/%	$FeSO_4 \cdot 7H_2O$/(g/L)	$ZnSO_4 \cdot 7H_2O$/(g/L)	Yeast extract %	RNA/ (mg/g DCW)
1	4	1	0.06	0.2	0.06	0.06	1	137.169
2	4	1	0.4	0.2	0.06	0.06	1	153.881
3	4	0.16	0.4	0.2	0.06	0.06	1	143.604
4	4	1	0.4	0.2	0.06	0.06	1	151.222
5	5	0.5	0.2	0.2	0.06	0.06	1	151.491
6	2.32	1	0.4	0.2	0.06	0.06	1	130.947
7	5.68	1	0.4	0.2	0.06	0.06	1	138.723
8	4	1	0.4	0.2	0.06	0.06	1	159.956
9	3	0.5	0.2	0.2	0.06	0.06	1	153.189
10	5	0.5	0.6	0.2	0.06	0.06	1	161.920
11	5	1.5	0.6	0.2	0.06	0.06	1	145.244
12	4	1	0.4	0.2	0.06	0.06	1	152.288
13	4	1	0.4	0.2	0.06	0.06	1	147.688
14	5	1.5	0.2	0.2	0.06	0.06	1	132.271
15	3	0.5	0.6	0.2	0.06	0.06	1	148.562
16	4	1	0.4	0.2	0.06	0.06	1	151.243
17	3	1.5	0.6	0.2	0.06	0.06	1	132.192
18	4	1	0.74	0.2	0.06	0.06	1	151.409
19	4	1.84	0.4	0.2	0.06	0.06	1	136.391
20	3	1.5	0.2	0.2	0.06	0.06	1	133.535

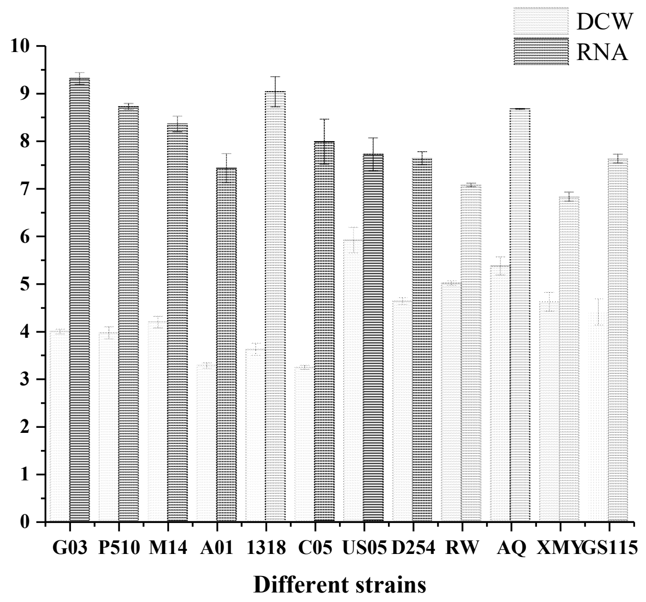

Figure S1. Comparison of ribonucleic acid and dry weight of different strains(G03, P510, M14, A01, 1318, C05, US05 were S. pastorianus strains, D254, RW were wine yeasts, AQ, XMY was baker's yeast and GS115 was a P. pastoris strain which all stored in author's laboratory). Data are the average of three independent experiments. Error bars represent ± SD.

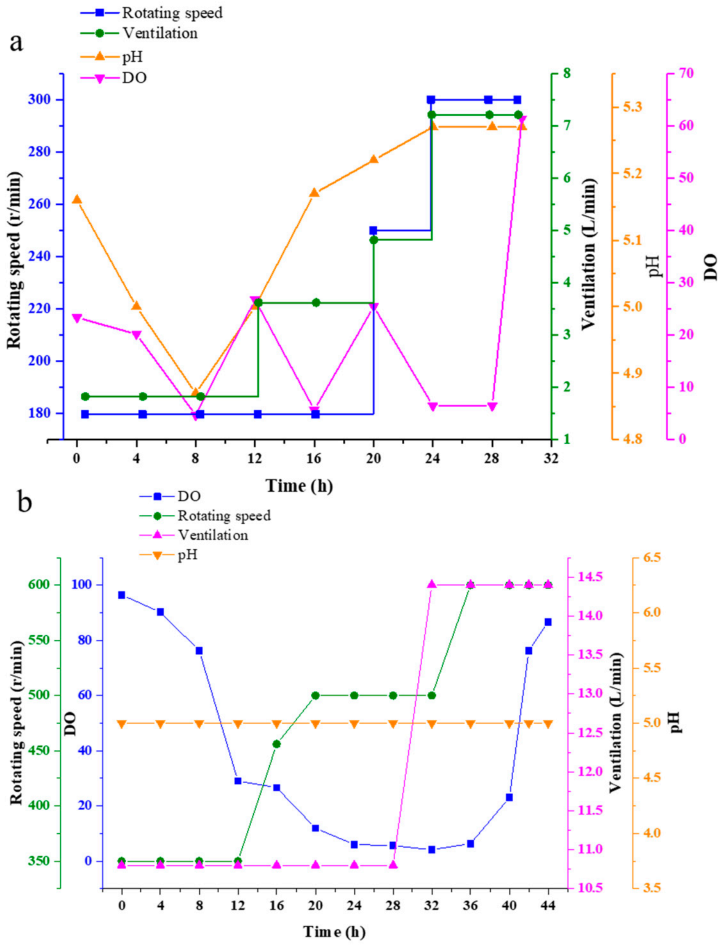

Figure S2. Conditions changes of batch (a) and fed-batch (b) fermentation in 5L fermenter.

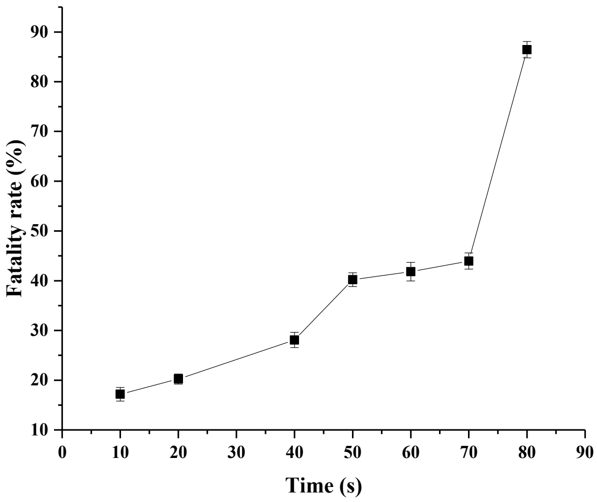

Figure S3. The relationship between lethality rate and time of mutagenesis. Data are the average of three independent experiments. Error bars represent ± SD.

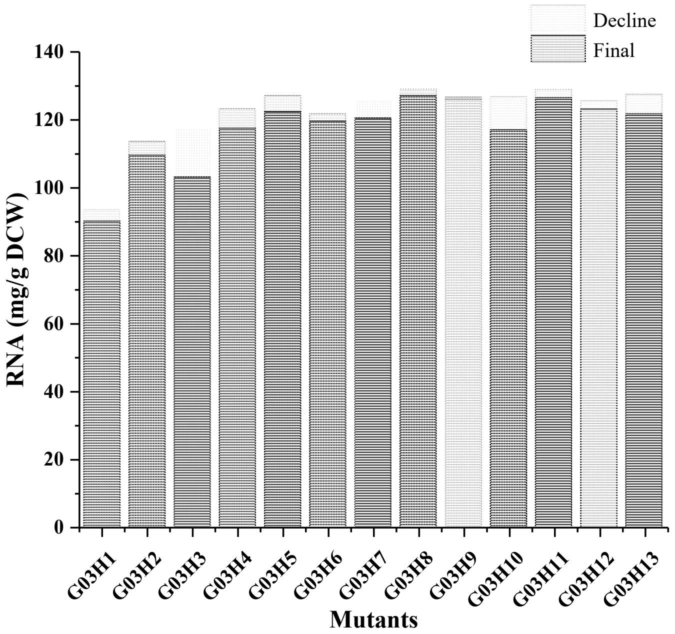

Figure S4. RNA content genetic stability of different mutants (Decline, decrease in RNA content after eight passages; Final, RNA content of mutants after eight passages).

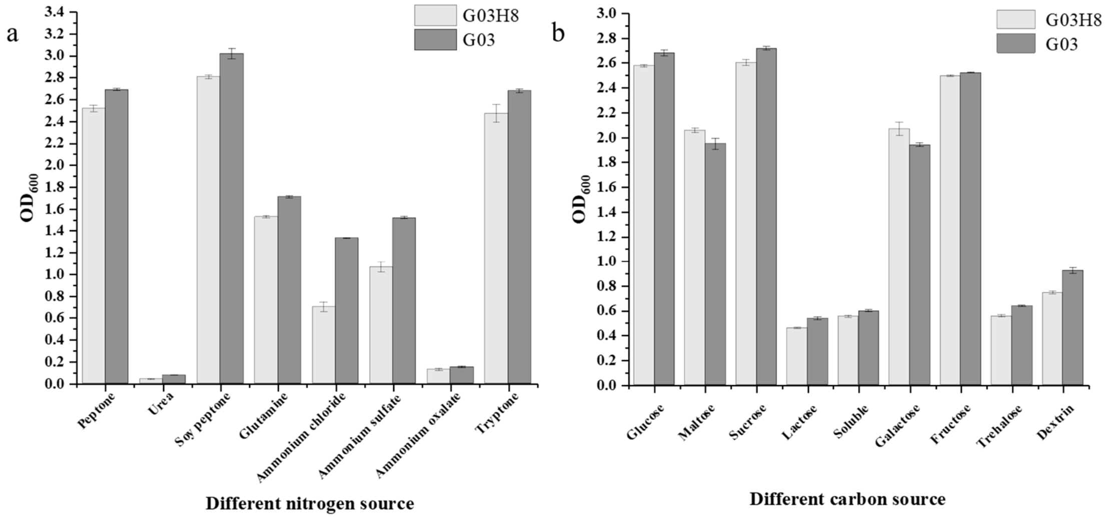

Figure S5. Growth of G03 and G03H8 on media with different nitrogen (a)and carbon (b)sources. Data are the average of three independent experiments. Error bars represent ± SD.

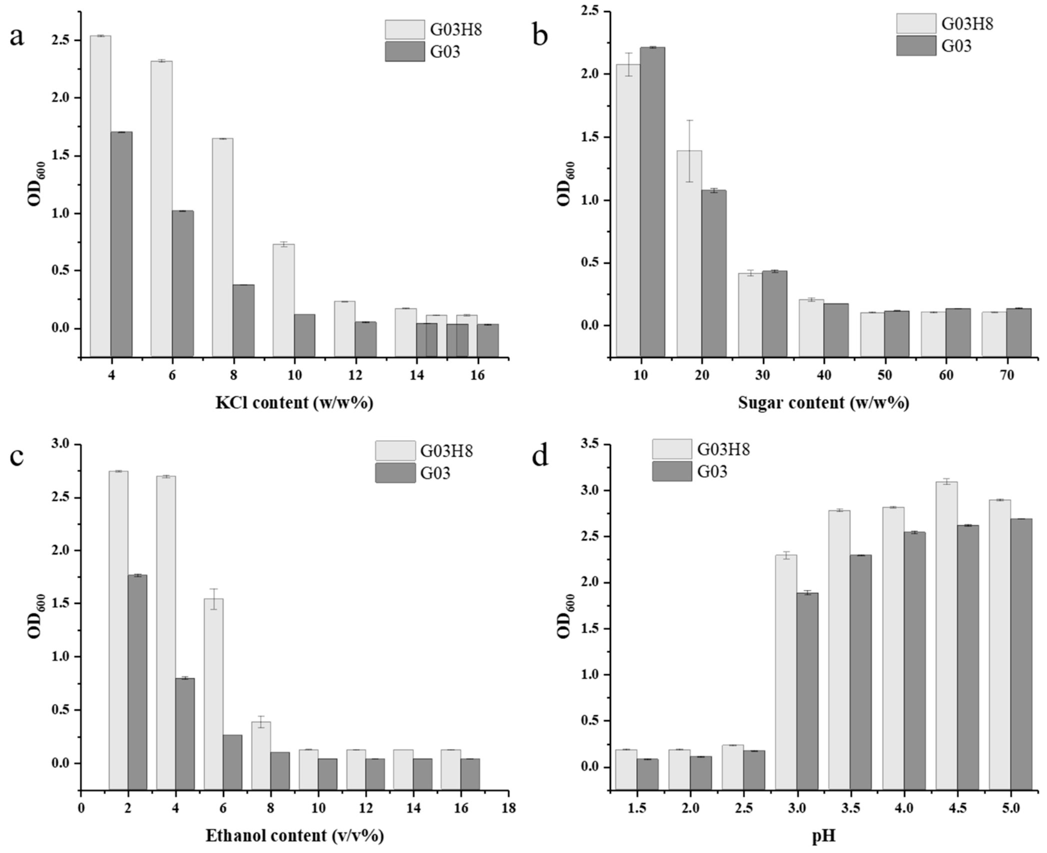

Figure S6. Environmental tolerance comparison of G03 and G03H8((a)Potassium chloride tolerance; (b) Sugar tolerance; (c) Ethanol tolerance; (d)Acid resistance). Data are the average of three independent experiments. Error bars represent ± SD.
